# Charting the landscape of microbiota in bladder cancer: Molecular mechanisms and therapeutic implications

**DOI:** 10.1016/j.gendis.2026.102270

**Published:** 2026-06-02

**Authors:** Zhihang Luo, Daming Xu, Zizhuo Wang, Longxiang Wu, Chunlin Ou, Zhenyu Ou

**Affiliations:** a Department of Urology, Xiangya Hospital, Central South University, Changsha, Hunan 410008, China; b State Key Laboratory of Oncology in South China, Guangdong Provincial Clinical Research Center for Cancer, Sun Yat-sen University Cancer Center, Guangzhou, Guangdong 510060, China; c Department of Pathology, Xiangya Hospital, Central South University, Changsha, Hunan 410008, China; d National Clinical Research Center for Geriatric Disorders, Xiangya Hospital, Central South University, Changsha, Hunan 410008, China

**Keywords:** Biomarker, Bladder cancer, Microbiota, Microenvironment, Therapy

## Abstract

The microbiota refers to the assemblage of microorganisms present in a particular environment, which can maintain homeostasis and modulate immune function in the human body. Microbiota dysbiosis disrupts bodily functions, leading to a series of human diseases, including cancer. Recent studies have suggested that tumor microbiota play important roles in the tumorigenesis of bladder cancer (BLCA), including malignant proliferation, invasion and metastasis, chemo-radiotherapy resistance, and immune response. In this review, we systemically classify the microbiota of the digestive and urinary systems associated with BLCA, as well as the intratumoral microbiota within BLCA tissue. This review provides a comprehensive summary of the characteristics of these microbial populations and explores their potential roles in the development, detection, diagnosis, and treatment of BLCA, with a focus on immunotherapy, while also discussing existing problems and possible development directions. Given the complexity and breadth of this field, clinicians should carefully consider the influence of the microbiota throughout the entire diagnostic and therapeutic process for BLCA.

## Background

Bladder cancer (BLCA) is the 11th most common cancer worldwide.^1^ As one of the most common tumors of the urinary system, there are more than 570,000 new cases of BLCA each year worldwide and more than 210,000 related deaths.^2^ BLCA can be divided into two categories: non-muscular-invasive BLCA (NMIBC) and muscular-invasive BLCA (MIBC). In NMIBC, according to the pathological tumor–node–metastasis staging system, tumors are confined to the mucosa and submucosa, and include carcinoma *in situ*, pTa and pT1 tumors, whereas in MIBC, tumor cells invade the muscular layer, and include pT2–pT4 tumor stages.^3^ Although approximately 75% of patients with BLCA have NMIBC at first diagnosis, more than 60% experience disease recurrence within two years of standard treatment, and 10%–20% of these patients progress to MIBC.^4^ NMIBC is treated with transurethral resection and intravesical therapies such as chemotherapy or Bacillus Calmette-Guérin (BCG), although it has a 60% 1-year relapse rate. MIBC is typically managed with neoadjuvant chemotherapy, radical cystectomy, and pelvic lymph node dissection.^5^ Radical cystectomy poses high risks and perioperative mortality, especially in older patients.^6^ Complications include infections, fistulas, and deep vein thrombosis, with 49% of MIBC patients choosing bladder preservation therapies.^7^ Notably, in recent years, the rise of immunotherapy and antibody–drug conjugates has continuously reshaped the treatment landscape for BLCA.^8^, ^9^, ^10^ However, the exact efficacy and recurrence indicators are still under investigation.

Cystoscopy is recommended for the preoperative diagnosis or regular postoperative examination of patients with symptomatic NMIBC. In particular, the first cystoscopy is advised in the 3rd month after surgery, and regular cystoscopy is recommended until the 5th year after surgery.^4^ Cystoscopy is an invasive procedure that cannot be tolerated by some patients. Additionally, the high economic burden resulting from frequent cystoscopy is a major factor contributing to the lower-than-anticipated frequency of cystoscopy.^11^ Owing to its high specificity, urine exfoliative cytology is strongly recommended and widely used as an auxiliary examination for cystoscopy to detect high-grade BLCA.^4^ However, the number of cells obtained may be limited, and urinary tract infection, calculi, and intravesical perfusion can influence the detection rate. The low sensitivity of cytology suggests that it cannot replace or reduce the need for cystoscopy. Over the past two decades, various biomarkers and advanced scoring systems have emerged as valuable non-invasive auxiliary tests for the preoperative diagnosis and postoperative monitoring of cancer,^12^, ^13^, ^14^ examples include nuclear matrix protein 22, bladder tumor antigen, UroVysion (using fluorescence *in situ* hybridisation), ImmunoCyt/uCyt+, prognostic immunotherapy score, and the cachexia index.^15^, ^16^, ^17^ Certain tests have been approved by the US Food and Drug Administration and European Medicines Agency for commercial urine biomarker testing. The emerging BLCA biomarkers ADXBLADDER and Bladder EpiCheck have also been reported to have comparable sensitivity and specificity;^18^ however, these markers require further study and verification in prospective randomized trials of low- and high-grade tumors. Hence, the identification of new therapeutic targets and strategies for BLCA is important.

The microbiota refers to the assemblage of microorganisms present in a particular environment. The term was initially defined by Lederberg and McCray,^19^ who emphasized the significance of microorganisms inhabiting the human body in health and disease. The study of microbiota mainly depends on the analysis of 16S rRNA genes, 18S rRNA genes, other marker genes, and genomic regions amplified and sequenced from a given biological sample. Taxonomic assignment can be performed using various tools to assign each sequence to microbial taxa (bacteria, archaea, eukaryotes, etc.) at different taxonomic levels, ranging from the phylum to species. The microbiome refers to the genomes of microorganisms and their surrounding environment. This definition is based on the “biome”, which encompasses all biological and microbial factors in the environment.^20^ The Human Microbiome Project is a recent research initiative that aims to encode the human microbiota and analyze its role. However, the urinary microbiota was not examined in the Human Microbiome Project. For a long time, the bladders of healthy individuals were considered sterile. This concept of a sterile bladder has been challenged by the discovery of resident bacteria.^21^ Rodriguez et al^22^ detected bacteria in bladder tumor tissues and found that tumors were almost entirely colonized by *Proteobacteria* (96%), which accounted for 93% of the total reads. However, *Stenotrophomonas maltopia* was the most abundant species (61% of total reads).

In this review, we summarize the relationship between the microbiota and BLCA, analyze the impact of the microbiota on BLCA immunotherapy, and discuss some current issues. In conducting this review, we aim to provide a potential theoretical foundation for targeted therapy in BLCA.

## Tumor microbiota

### Definition, characteristics, and history of tumor microbiota

The discovery and definition of the tumor microbiota have evolved progressively over time (Fig. 1). Tumor microbiota refers to the microbial communities, including bacteria, viruses, and fungi, present in the tumor microenvironment (TME). These microbiotas are closely associated with the occurrence, development, diagnosis, prognosis, and treatment of cancer.^23^ Nejman et al^24^ identified unique microbiota in different tumors, notably a rich diversity in breast cancer, and found correlations with tumor types, smoking status, and immunotherapy responses. The history of tumor microbiota dates back to around 1550 BCE, when the Egyptian physician Imhotep treated tumors by incising swellings to cause infections.^25^ In the 13th century, Peregrine Laziosi developed a massive tumor on his tibia, which led to severe infection after amputation; however, his cancer never recurred.^26^ Subsequent reports have indicated spontaneous regression of tumors after infection, and this rudimentary form of cancer immunotherapy was widely recognized and accepted until the 18th and 19th centuries. It was not until the late 19th century that William Coley successfully injected a vaccine made from two inactivated bacteria (*Streptococcus pyogenes* and *Serratia marcescens*) directly into tumors to treat patients with sarcoma. This was promoted as a classic example of immunotherapy and was widely disseminated.^23^^,^^25^^,^^27^

**Figure 1:**
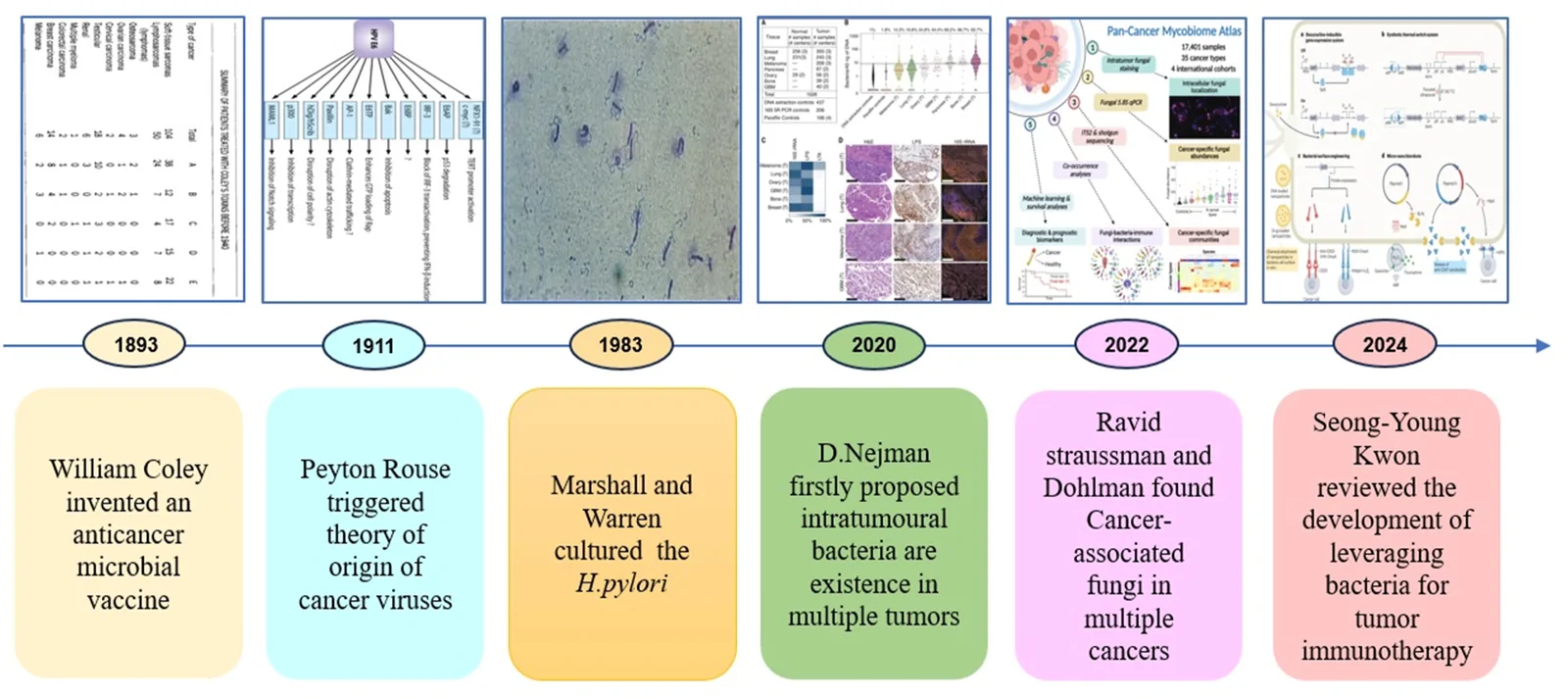
A historical timeline of the tumor microbiota. The milestones in the study of tumor microbiota and the main achievements of anti-tumor therapy with microbiota as the core are reviewed retrospectively.

In the 1900s, Thomas Glover and Virginia Livingston-Wheeler^23^^,^^28^ claimed that bacteria could grow from tumors and proposed a bacterial origin for cancer; however, their theory was ultimately proven incorrect. In 1911, Peyton Rous^29^ discovered that filtrates from chicken breast tumors could cause contagious sarcomas, initiating the theory of viral origins of cancer. Over the following decades, viruses capable of inducing cancer have been discovered, such as the Epstein–Barr virus (EBV), Kaposi's sarcoma-associated herpesvirus, human papillomavirus (HPV), human T-cell lymphotropic virus, hepatitis B virus, hepatitis C virus, and Merkel cell polyomavirus. In 1983, Marshall and Warren cultured *Helicobacter pylori* and demonstrated its role in peptic ulcers.^30^^,^^31^ Studies have linked this bacterium to gastric cancer, prompting research into bacteria's role in cancer. In the 21st century, advances in sequencing technology have revealed microbial communities in tumors, underscoring their significance in the TME and therapeutic outcomes.^32^, ^33^, ^34^, ^35^, ^36^, ^37^, ^38^, ^39^, ^40^, ^41^ Next-generation sequencing technology has significantly advanced the study of microbial communities in tumors. In 2020, Nejman et al^24^ conducted a comprehensive microbiota analysis of 1526 tumors and adjacent normal tissues across seven cancer types, including breast, lung, ovarian, pancreatic, melanoma, bone, and brain tumors, revealing insights into the spatial distribution of microbial communities within tumors. In 2022, Straussman et al^42^ found the distribution of fungi in 35 types of cancer, as well as their localization within cells, and their synergistic interactions with bacteria. Concurrently, Dohlman et al^43^ analyzed The Cancer Genome Atlas (TCGA) data, discovered fungi associated with gastrointestinal, lung, breast, and head and neck cancers, and studied the role of fungal DNA in diagnosis and prognosis. In the past decade, notable advances have been made using bacteria as tumor immunotherapy agents. In 2024, Kwon et al^44^ summarized data from multiple clinical trials on microbiota for cancer treatment, presented bacterial engineering strategies, highlighted the potential of bacteria in cancer therapy, and established a foundation for developing microbial agents in this field.

### Classification methods for tumor microbiota

The differences in microbial communities in different types of tumors and their contributions to maintaining homeostasis and participating in disease processes have become widely studied.^45^, ^46^, ^47^, ^48^, ^49^, ^50^

Based on their distribution, microbiota can be broadly classified into skin, oral, gastrointestinal, and urinary tract tumor microbiota. A study by Yang et al^51^ revealed that an increase or decrease in certain skin microbial species may be related to inflammatory responses and cellular damage, thereby affecting the development of skin cancer. In a study by Stasiewicz et al,^52^ specific oral tumor microbiota were identified, such as *Porphyromonas gingivalis* and *Streptococcus* species, as well as viruses such as HPV, human herpesvirus 8, and EBV, along with yeasts. The microbiota is closely associated with oral cancer occurrence and progression. Furthermore, research has indicated that members of the oral microbiota are associated with esophageal, gastric, pancreatic, colorectal, and lung cancers. Studies have indicated that *Helicobacter pylori* is one of the most important carcinogenic bacterial species in gastric cancer. Wu et al^53^ demonstrated that the gastric mucosal microbiota of patients with gastric cancer differed significantly from that of healthy individuals, with a marked increase in *Helicobacter pylori* in gastric cancer tissues. In addition, there have been numerous studies on the tumor microbiota in colorectal cancer. Fukuoka et al^54^ analyzed the microbiota in colorectal cancer tissues through 16S rRNA sequencing and identified *Bacteroides* and *Bifidobacterium*. Many studies have reported that the urinary microbiota in patients with BLCA, prostate cancer, and renal cell carcinoma show specific dominant bacterial genera, such as *Fusobacterium*, *Streptococcus*, and *Enterobacteriaceae*, compared with healthy individuals, indicating that these genera may be related to the occurrence and development of urological system tumors.^28^ In recent years, research on microbiota within tumors has increasingly gained widespread attention, with potential sources mainly including the digestive tract, respiratory tract, urinary tract, adjacent normal tissues, circulatory system, and accompanying tumor metastasis.^55^

### Role of microbiota in tumor development

Growing evidence suggests that various tumors are closely related to microbial infections.^32^^,^^38^ Recognizing the inseparable relationship between microbiota, the immune system, and tumors, microbiota can indirectly promote tumor occurrence and metastasis by suppressing immune responses. Yang et al^51^ have reported that microbiota can regulate the occurrence and development of tumors through three mechanisms: inducing DNA mutations, activating oncogenic signaling pathways, and shaping the inflammatory TME.

### Inducing DNA damage and mutations

Many pathogenic microbiotas have evolved compounds that cause DNA damage, cell-cycle arrest, and genetic instability.^56^ Genotoxins can cause DNA damage and chromosomal instability or impair DNA repair in cells. Certain microbiota in the TME produce these substances, increasing DNA mutations in colonized tissue, which can lead to uncontrolled cell growth and tumor development. Approximately 60% of colorectal cancer samples contain pks ^+^ *E. coli* strains, which produce genotoxins and proteins linked to a large genomic island called pks.^57^ In addition, an imbalance in the DNA damage response system can increase DNA damage and carcinogenesis. Viruses can easily cause DNA damage, thereby manipulating the DNA damage response system to guide cancer development. For example, toxins from human T-lymphotropic virus type 1 and high-risk HPV oncoproteins can suppress the DNA damage response system, promoting carcinogenesis and the formation of malignant tumors.^58^^,^^59^ Notably, whether the DNA damage caused by pathogenic microbiota is sufficient to cause cancer still requires extensive research.

### Activating oncogenic signaling pathways

Signaling pathways influence essential activities such as cell movement, survival, and metabolism in both normal and tumor tissues. Activation of these pathways, particularly those related to cell survival, growth, and proliferation, such as the PI3K/Akt, JAK–STAT, and Wnt/β-catenin pathways, plays a crucial role in tumor development. A recent study in patients with lung cancer has shown that the up-regulation of PI3K signaling pathway is related to the enrichment of oral microbiota, such as *Porphyromonas* and *Streptococcus*, in the lower respiratory tract.^60^ *In vitro* experiments have also shown that airway epithelial cells exposed to *Porphyromonas*, *Prevotella*, and *Streptococcus* exhibit up-regulation of the PI3K signaling pathway.^60^ The accumulation of oral commensal bacteria in the lower respiratory tract may lead to the up-regulation of the PI3K pathway, thereby promoting the occurrence of lung cancer.^56^ The JAK–STAT signaling pathway plays a crucial role in almost all cytokine-driven signal transduction and controls cell-cycle progression and apoptosis.^61^ Chattopadhyay et al^62^ have demonstrated that *Porphyromonas gingivalis* activates the JAK1/STAT3 and PI3K/Akt signaling pathways, inhibiting chemically-induced intrinsic mitochondrial apoptosis in gingival epithelial cells. This bacterium is commonly found in subgingival plaques and is abundant in malignant oral epithelial cells, potentially linked to squamous cell carcinoma of the gingiva.^63^^,^^64^ The Wnt/β-catenin signaling pathway regulates stemness, polarity, and growth, and its abnormal activity can lead to oncogene transcription and tumor progression.^65^ *Helicobacter pylori* infection causes gastric cancer, with some strains expressing the cytotoxin-associated gene A protein, which induces gastric mucosal epithelial cell carcinogenesis by regulating β-catenin, thereby promoting tumor progression.^66^^,^^67^ The extracellular signal-regulated kinase (ERK) signaling pathway, commonly known as the Ras–Raf–MEK–ERK signaling cascade, is also involved in tumor occurrence. Kim et al^68^ have reported that microbiota can directly or indirectly activate ERK signaling in tumor cells, thereby regulating cell growth and proliferation to affect tumor occurrence.

### Shaping the TME

The TME refers to the internal environment in which tumor cells live, and includes immune cells, cancer-associated fibroblasts, tumor microbiota, microbial products, cytokines, chemokines, and the extracellular matrix.^69^ The TME is crucial in cancer pathogenesis, influencing tumor development and treatment responses, especially through an inflammatory microenvironment. In the late 19th century, German pathologist Virchow^70^ proposed that inflammation promoted cancer occurrence. Many clinical and evidence-based medical studies^71^ have shown that chronic inflammation is a risk factor for various tumors, especially digestive tract tumors, such as esophageal, gastric, liver, and colorectal cancers. Cutolo et al^72^ found that abnormal transcription of genes for inflammatory mediators, growth factors, and angiogenic factors linked to inflammation and cancer. While *Helicobacter pylori* infection is a major pathogenic factor for gastric cancer, it lacks direct genotoxicity, and so it is believed to indirectly lead to cancer by triggering a persistent inflammatory response.^73^

Microbiota plays a significant role in the TME by directly interacting with tumor cells, regulating immune cell functions, and reshaping the environment through metabolic products, influencing tumor occurrence and progression.^74^, ^75^, ^76^ Zhu et al^75^ have demonstrated that some bacteria within tumors can reorganize the actin cytoskeleton of cancer cells, thereby enhancing their migratory ability. Microbial-derived metabolites, such as inosine, bile acids, and enzymes, can influence cancer progression by affecting the TME. Microbially derived bile acids have been proven to regulate late natural killer cells and play an important role in the occurrence and progression of liver cancer.^77^ Azurin secreted by *Pseudomonas aeruginosa* can induce tumor cell apoptosis, whereas aldolase A released by cancer cells can promote the adhesion and colonization of cancer cells by *P. aeruginosa*.^78^^,^^79^ In addition, microbiota can suppress anti-tumor immune responses by affecting immune cells in the TME, such as regulatory T cells (Tregs) and myeloid-derived suppressor cells, promoting tumor development.^74^^,^^80^ In addition to suppressing anti-tumor immunity, microbiota can enhance anti-tumor immunity. Oncolytic viruses or bacteria can specifically target the TME, lyse tumor cells, and induce anti-tumor responses.^81^^,^^82^

### Detection techniques for tumor microbiota

Recently, with the development of various detection technologies, many previously undiscovered microbiotas have been identified, and their association with the occurrence and development of tumors has been explored. Commonly used detection methods include 16S rRNA gene sequencing, metagenomic sequencing, deep sequencing based on universal marker gene amplicons, transcriptome sequencing, and metabolome sequencing,^83^^,^^84^ which are analyzed through microbiota data analysis platforms and tools, such as QIIME 2, Mothur, MetaPhlAn, PICRUSt, MaAsLin 3, and MicrobiomeAnalyst, helping to identify the composition of microbiota.^85^^,^^86^ We summarized the main detection methods for tumor microbiota currently available, along with their advantages and disadvantages (Table 1).^24^^,^^75^^,^^87^, ^88^, ^89^, ^90^, ^91^, ^92^, ^93^, ^94^, ^95^, ^96^, ^97^, ^98^, ^99^, ^100^, ^101^, ^102^, ^103^, ^104^, ^105^, ^106^

**Table 1:** The advantages and disadvantages of the main detection methods on tumor microbiota.

Detection methods	Definition	Advantage	Disadvantage	Microbial type	Reference
16S rRNA sequencing	Sequencing of highly variable 16S rRNA regions allows bacterial composition to be classified.	Fast, inexpensive, and able to detect bacterial communities at the species and strain levels	Limited precision, poor repeatability, inability to detect non-bacterial microbiota, and potential host genome contamination.	Bacteria and archaea	87, 88, 89
IS-pro	Distinguish bacterial species by 16S–23S rDNA spacer length; classified with fluorescent-labeled PCR primers.	Fast, easy to operate, and capable of detecting bacterial communities at the species level.	Patent	Bacteria and archaea	90,91
Metagenomic sequencing	Sequence the total genetic material of all microbiota in a sample to study community structure and function.	High resolution, broad application, and functional analysis are possible.	Expensive, complex data analysis; extensive computation; time-consuming; potential host genome contamination.	Bacteria, fungi, viruses, and other microbiota	92, 93, 94, 95
Metatranscriptomics	Qualitative and quantitative analysis of microbial RNA transcription in samples.	Reflects gene expression and aids in understanding microbial function and mechanisms.	Expensive, short RNA half-life, sensitive to host RNA.	Bacteria, fungi, viruses, and other microbiota	96, 97, 98
Proteomics	Qualitative and quantitative analysis of all proteins in tissues or cells.	Reveals interactions between microbes and host cells, and carcinogenesis mechanisms.	Insufficient sensitivity and accuracy in protein expression detection; strict analysis conditions are required for certain proteases.	Bacteria, fungi, viruses, and other microbiota	94,98
Metabolomics	Qualitative and quantitative analysis of all metabolites of microbiota in samples.	High resolution; enables *in situ* analysis and constructs metabolic networks.	Unable to observe dynamically.	Bacteria, fungi, viruses, and other microbiota	75,98,99
FISH	Use fluorescently labeled nucleic acid probes to specifically bind target DNA or RNA, directly detecting specific nucleic acid molecules in cells.	Visualize microbial distribution, high sensitivity, and strong specificity.	Requires specific probes, complex operation, and high technical requirements.	Specific types of microbiota	98,100
qRT-PCR	Method for qualitative or quantitative analysis of initial templates by monitoring real-time accumulation of PCR product fluorescence signals.	High sensitivity, strong specificity, short operation time, and accurate quantification.	Requires known target sequences and cannot detect unknown microbiota.	Various known microbiota	101,102
IHC	Locate antigens in cells using antigen–antibody binding and chemical color reactions.	Visualize microbial presence and distribution, precise localization, and high specificity.	High false positive rate, possible cross-reactions.	Specific types of microbiotas	24,100
Culture	Identify and count microbiota by culturing them in suitable media to grow and reproduce.	Obtain live microbiota suitable for antibiotic susceptibility testing.	Limited to cultivable microbiota, time-consuming, and potential contamination.	Cultivable microbiota	87,98,103
Nucleic acid hybridization	Analyze specific nucleic acids by hybridizing labeled probes and detecting signals.	High sensitivity and specificity.	Complex operation.	DNA viruses, RNA viruses, and viroids	104, 105, 106

Tumor microbiota detection and analysis involves several steps (Fig. 2): sample collection from tumor tissues or surrounding environments (such as feces, urine, and oral cavity); DNA/RNA extraction from samples; 16S rRNA or metagenomic sequencing; data processing and analysis, with data analysis platforms such as QIIME 2 and Mothur used for quality control, OTU clustering (for 16S rRNA data) or species annotation (for metagenomic data), and other sequencing data processing; functional analysis, using tools such as PICRUSt, to predict the functional potential of microbial communities; statistical analysis of the relationship between microbiota data and tumor status or treatment response; and result interpretation and reporting.

**Figure 2:**
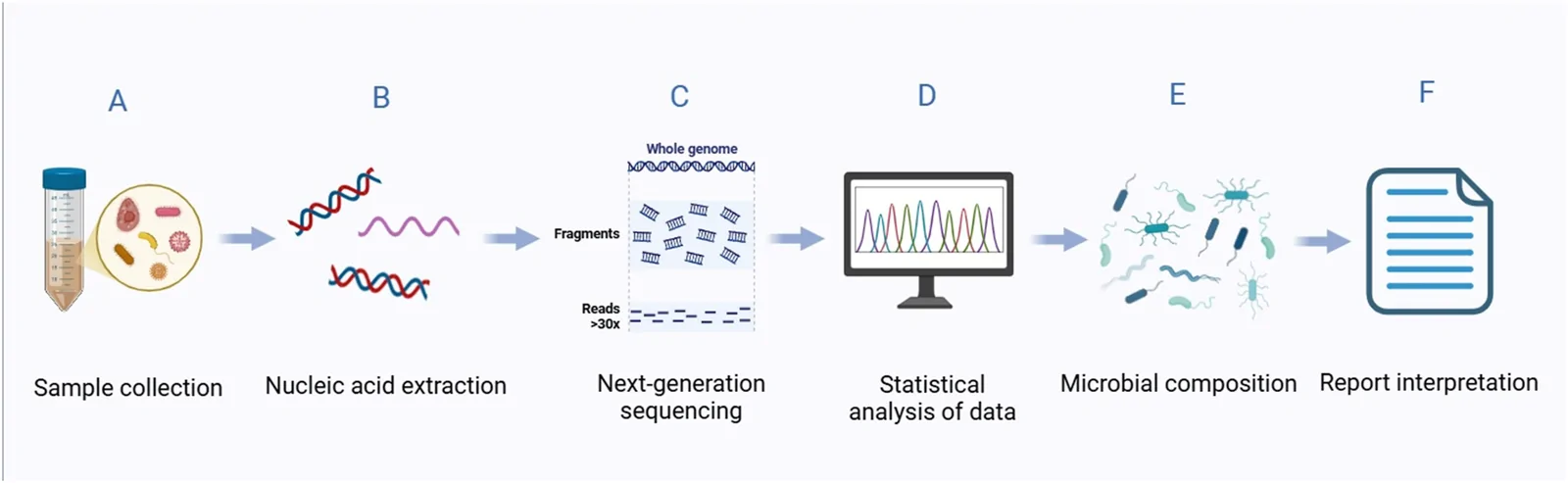
The detection process of tumor microorganisms was briefly summarized.

### Microbiota in BLCA

Microbiota represents an essential component in BLCA. Recent research has indicated the presence of a unique microbiota in urine, with certain microbial functional activities closely associated with BLCA.^107^^,^^108^ Additionally, numerous studies indicate that both urinary and gut microbiota significantly influence the occurrence and progression of BLCA through various pathways.^109^

### Relationship between urinary microbiota and BLCA

A prospective study by Bi et al^110^ analyzed urine samples from 29 patients with BLCA and 26 controls, finding significant differences in five bacterial genera: *Streptococcus*, *Bifidobacterium*, *Lactobacillus*, *Veillonella*, and *Actinomyces*. *Actinomyces* was more abundant in patients with BLCA, and the α diversity of the microbiota was higher in the BLCA group compared with controls. Viral infections may also be a cause of bladder cancer, although current research is limited. A cohort study by Hrbáček et al^107^ investigated the association between common DNA viruses in urine and BLCA, and found a significant difference in HPV presence between patients with BLCA and the control group, indicating a potential link between HPV and the occurrence of BLCA. Similarly, EBV^111^ and cytomegalovirus^112^ infections have been implicated in the risk of BLCA based on Mendelian randomization and colocalization analyses. Additionally, the detection rates of herpes simplex virus (HSV) types 1 and 2 in bladder cancer are similar to those in inflammatory or normal tissues, while EBV is found in a significant proportion of BLCA cases.^113^^,^^114^

### Relationship between gut microbiota and BLCA

Multiple observational studies have indicated differences in the composition and diversity of the gut microbiota between healthy individuals and patients with BLCA. In a case–control study conducted in Harbin, China, He et al^108^ observed a lower abundance of *Prevotella* in the intestines of patients with BLCA. Similarly, Yin et al^115^ demonstrated that specific gut microbiota, such as *Rikenellaceae* and *Allisonella*, were significantly associated with BLCA, and sensitivity analyses confirmed the reliability of the results. These studies preliminarily reveal a potential causal relationship between gut microbiota dysbiosis and BLCA, providing a basis for future exploration of the specific mechanisms by which gut microbiota dysbiosis contributes to the occurrence and development of BLCA.

### Differences in microbiota between tumor and adjacent mucosa in BLCA

A study by Parra-Grande et al^116^ from Spain found that microbial richness was higher in non-tumor mucosal tissues of patients with BLCA, exhibiting greater α diversity, with the phylum *Actinobacteria* being significantly more enriched in non-tumor than in tumor mucosal tissues. Two enriched microbial clusters were identified in the tumor mucosal tissues: cluster 1, consisting of *Barnesiella*, *Parabacteroides*, *Prevotella*, *Alistipes*, and *Lachnospiraceae*, and cluster 2, consisting of *Staphylococcus*. Additionally, multivariate analysis revealed significant differences in microbial composition based on tumor grade, with low-grade tumors characterized by *Enterococcus* enrichment.

### Relationship between microbiota metabolites and BLCA

Microbiota can regulate pathological processes in BLCA through various means, including their derived metabolic products and the induction of inflammatory responses, affecting tumor occurrence, development, and treatment response. Indeed, Wang et al^117^ have indicated that the gut microbiota can exert an anti-cancer effect on bladder urothelial cells through its metabolic product, butyrate. Butyrate is a short-chain fatty acid that regulates the cell cycle, cell growth, apoptosis, and gene expression and is produced by *Butyricicoccus pullicaecorum*. The effect of butyrate on BLCA was verified through cell and animal experiments; butyrate significantly up-regulated GPR43, GPR109B, FABP4, and BLCAP (a tumor suppressor protein associated with apoptosis), reduced the expression of CDK1, and blocked the cell cycle at the G2/M phase, thereby promoting tumor apoptosis and inhibiting the proliferation of BLCA. In a comprehensive analysis of fecal metabolomics in a Chinese population, Mansour et al^118^ found that 17 metabolites, including undecenoic acid, 3-methoxytyrosine, abrine, o-aminobenzenesulfonic acid, and arachidonic acid, were significantly reduced in the feces of patients with BLCA, while cholesterol sulphate was significantly increased. These findings provide evidence for a potential causal link between the metabolic products of the gut microbiota and BLCA.

### Biological significance of microbiota in BLCA

The tumor microbiota plays crucial roles in the tumorigenesis of BLCA, contributing to malignant proliferation, invasion, metastasis, resistance to chemo-radiotherapy, and modulation of the immune response (Fig. 3).

**Figure 3:**
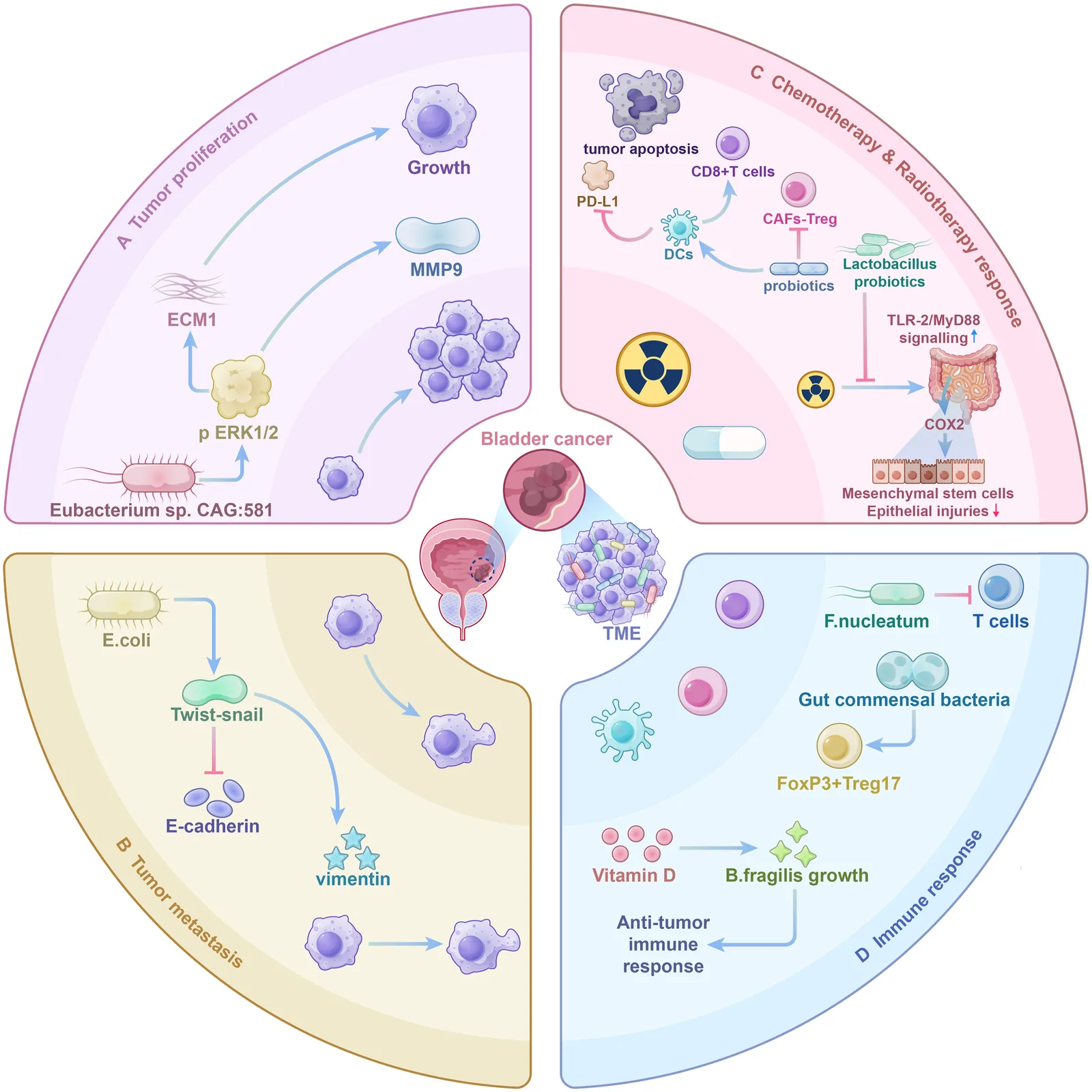
Relationship between microbiota and the malignant phenotype in BLCA. **(A)** Microbiota promote the malignant proliferation of tumors. For example, the urinary tract microbe *Eubacterium sp*. *CAG:581* promotes tumor cell proliferation by inducing phosphorylation of ERK1/2 protein in BLCA tissue, up-regulating ECM1 and MMP9 expression. **(B)** Microbiota accelerate tumor invasion and metastasis. Microbiota such as *E. coli* promote BLCA invasion and metastasis through the epithelial–mesenchymal transition pathway, activating epithelial–mesenchymal transition-related gene expression (*e.g.*, Twist, Snail, E-cadherin, Vimentin). **(C)** Microbiota affect chemo-radiotherapy resistance. Probiotics protect intestinal mucosa from radiation damage by stimulating mesenchymal stem cell migration to crypts via a TLR-2/COX-2-dependent manner. They also increase CD8^+^ T cell infiltration in tumor tissue, inhibit dendritic cell PD-L1 expression, enhance anti-tumor immune response, promote tumor cell apoptosis, and suppress immunosuppressive regulatory T cells (Tregs). **(D)** Microbiota interact with the tumor immune microenvironment. Microbiota such as *Fusobacterium nucleatum* and gut commensals induce an immunosuppressive tumor microenvironment to inhibit tumor immune responses. Studies show that vitamin D promotes the growth of “anti-cancer bacteria”, *Bacteroides fragilis*, which positively regulates anti-tumor immune responses and improves the efficacy of immune checkpoint inhibitors.

### Microbiota promotes the proliferation of BLCA

BLCA is a malignant tumor originating from the urothelium and is characterized by uncontrolled and unlimited proliferation of atypical cells. Many factors affect the malignant proliferation of BLCA, and numerous studies^119^, ^120^, ^121^, ^122^ have shown that microbiota plays an important role in this process. We speculate that the urinary microbiota may promote the progression of early-stage bladder cancer, but its specific mechanisms remain unclear due to confounding environmental factors.

Zhang et al^123^ conducted a metagenomic analysis of the urinary microbiota of 51 patients with NMIBC and 47 healthy controls, and identified a significantly different urinary microbe, *Eubacterium* sp. *CAG:581*. Under anaerobic conditions, the coculture of NMIBC organoids with uropathogenic *Eubacterium* sp. *CAG:581* accelerated the proliferation of NMIBC organoids, and differential gene expression analysis showed that extracellular matrix protein 1 (ECM1) and matrix metalloproteinase 9 (MMP9) were the most up-regulated genes induced by the phosphorylation of ERK1/2 in NMIBC organoids. This study indicated that the uropathogenic *Eubacterium* sp. *CAG:581* promoted the progression of NMIBC through the ECM1/MMP9 pathway and may serve as a promising non-invasive diagnostic biomarker for NMIBC.

### Microbiota affects the invasiveness and metastasis of BLCA

Invasiveness and metastasis are hallmarks of malignancy and involve the spread of tumor cells from the primary site to distant organs, contributing significantly to tumor mortality and poor prognosis. Clinical evidence indicates that MIBC exhibits high malignancy and metastatic potential, often progressing from NMIBC. Differences in microbiota composition between NMIBC and MIBC may influence tumor behavior. Hussein et al^124^ reported the differences in tumor microbiota among patients with NMIBC, patients with MIBC, and healthy controls and found that compared with the healthy control group, the β-diversity (differences in microbial communities between different samples) of the urinary microbiota in patients with BLCA was significantly different. *Actinomyces*, *Acinetobacter*, *Brevundimonas*, and *Brucella* were significantly enriched in the urine samples of patients with BLCA, and the abundance of *Haemophilus* and *Veillonella* was significantly increased in the urine of patients with MIBC, while *Pseudomonas aeruginosa* was significantly enriched in patients with NMIBC. Another study comparing microbiota in patients with NMIBC or MIBC found that microbial diversity was significantly higher in NMIBC tissues. Notably, *Ralstonia_sp000620465* was more abundant in MIBC, suggesting its potential link to invasiveness. NMIBC was enriched with species such as *Acinetobacter guillouiae*, *Anoxybacillus arupiensis*, *Brevibacillus agri*, and *Staphylococcus lugdunensis*, while MIBC showed higher levels of *Ralstonia mannitolilytica*, *Ralstonia pickettii*, and *Ralstonia_sp000620465*,^125^ suggesting a potential link between microbiota dysbiosis and the progression of BLCA. Li et al^122^ explored the association between microbial abundance in the microbiota of MIBC and the epithelial–mesenchymal transition and found that microbiota such as *E coli*, butyrate-producing bacteria SM4/1, and *Spirulina* were related to the expression of the epithelial–mesenchymal transition-related genes E-cadherin, vimentin, SNAI2, SNAI3, and TWIST1. The microbial abundance significantly correlated with the expression of genes encoding extracellular matrix proteins, particularly collagen and elastin. These findings suggest that microbiota plays an important role in the regulation of the epithelial–mesenchymal transition in MIBC, thereby promoting the invasion and metastasis of BLCA.

### Impact of microbiota on BLCA response to radiotherapy and chemotherapy

Radiotherapy and chemotherapy are standard treatments for advanced and metastatic tumors. In BLCA, resistance arises from multiple factors, and understanding the underlying molecular mechanisms is critical. The microbiota and its metabolites are closely related to treatment efficacy and side effects. Yang et al^126^ indicated that certain microbial metabolites may have contradictory effects, depending on the type of cancer. Furthermore, the metabolic effects of microbes on certain anti-cancer drugs can lead to drug ineffectiveness and exacerbation of adverse reactions. Miyake et al^127^ investigated the relationship between the gut microbiota and chemotherapy in urothelial carcinoma, finding that chemotherapy significantly altered gut microbiota composition. Their animal experiments demonstrated that combining chemotherapy with oral probiotics significantly increased the proportion of CD8^+^ T cells and tumor cell apoptosis in tumor tissue, while reducing PD-L1 expression in dendritic cells. This suggests that oral probiotics can enhance anti-tumor immunity and promote apoptosis, potentially benefiting patients with advanced urothelial carcinoma undergoing chemotherapy.

### Microbiota, immune microenvironment, and inflammation in BLCA

A recent study^128^ has indicated that the microbiota of patients with BLCA is closely related to their immune microenvironments. Microbiota can influence the progression of BLCA and treatment responses by modulating innate and adaptive immune responses. In addition, the gut microbiota and its metabolites regulate the functions of immune cells in the TME, such as macrophages, myeloid-derived suppressor cells, and Tregs, thereby influencing anti-tumor immunity and treatment responses.^129^ Nardelli et al^130^ have reported that certain microbiota can promote the occurrence and progression of cancer by inducing inflammatory responses. For example, *Fusobacterium nucleatum* is a Gram-negative anaerobic oral symbiont that triggers chronic inflammatory responses and promotes the occurrence and development of various cancers, including breast cancer. The presence of *F. nucleatum* samples has been detected in BLCA tissue.^131^ However, the specific role of *F. nucleatum* in BLCA development and its effect on the TME and immune responses requires further investigation. Additionally, certain species in the urinary microbiota can activate host immune cells through the metabolic product lipopolysaccharide, inducing chronic inflammatory responses,^132^ thereby promoting the proliferation and metastasis of BLCA. Fidelle et al^133^ investigated the influence of the gut microbiota on cancer immunotherapy by regulating immunosuppressive intestinal T cells and found that gut symbionts could induce the differentiation of immunosuppressive FOXP3^+^ Tregs (Treg17). The interaction between α4β7 integrin in the gut and its ligand MAdCAM-1 maintained the positioning of Treg17 cells in the gut. The authors suggest that disrupting the MAdCAM-1-α4β7 interaction may lead to the migration of these cells from the gut to the tumor, thereby weakening the immune response to tumor immunotherapy.

## Clinical significance of microbiota in BLCA

### Diagnostic value of microbiota in BLCA

Microbiota that participates in the occurrence and development of BLCA may serve as tumor biomarkers (Table 2).^108^^,^^110^^,^^116^^,^^119^^,^^123^^,^^124^^,^^134^, ^135^, ^136^, ^137^, ^138^ Advancements in microbial sequencing technology have enhanced the diagnosis, classification, grading, and staging of tumors. While the causal relationship between microbiota and BLCA remains unclear, there are two potential hypotheses. The first suggests that specific microbiota may accumulate in the bladder or intestine, promoting the onset and progression of BLCA; the second posits that BLCA develops first, creating a unique intestinal or urinary environment that supports the survival of specific microbiota, leading to their enrichment. Regardless of which hypothesis is accurate, the identification of characteristic microbiota strongly correlated with BLCA can serve as valuable biological markers for risk assessment of BLCA. A study by Chipollini et al^119^ showed that the urine microbial community diversity was significantly reduced in patients with BLCA compared with those without BLCA, and *Bacteroides* and *Enterococcus faecalis* were significantly enriched in the urine of patients with MIBC, whereas no meaningful bacterial taxa were found in the NMIBC group. Another study that included 43 patients with BLCA and 10 healthy controls^124^ found significant differences in β diversity between the two groups. The β-diversity of *Actinomyces*, *Achromobacter*, *Bacillus brevis*, and *Brucella* in the urine of those with BLCA was significantly increased, while *Hemophilus* and *Vickerella* were significantly higher in the urine of those with NMIBC or MIBC than in the control group. The amount of *Cupriavidus* in the urine of the NMIBC group was significantly higher than that in the urine of the control group, confirming differences in the microbiota between different subtypes of BLCA. Summarizing the characteristic microbiota of different BLCA subtypes can inform clinical treatment decisions, including surgical method selection and the need for neoadjuvant therapy. Bioinformatics analysis^42^ of microbial sequences from the TCGA dataset was conducted to investigate the bacterial and fungal characteristics of various tumors. It is important to note that the TCGA dataset does not include one major BLCA subtype, which limits its representativeness. Nevertheless, microbiota can serve as a biomarker and have potential as a non-invasive tool for auxiliary diagnosis of early BLCA, owing to simple detection methods and decreasing costs. This underscores its significant value in early tumor diagnosis.^139^ While specific microbiota cannot currently replace urinary exfoliation cytology in clinical testing, combining characteristic microbiota with other biological markers as a “biomarker set” may enhance the sensitivity and specificity of non-invasive assays. Further randomized clinical trials are needed to determine the cost-effectiveness and its influence on patient choice.

**Table 2:** Clinical value of bladder cancer-related microbiota.

Country	Sampling type	Total size	Sample size				Results		Reference
			NMIBC (*n*)	MIBC (*n*)	Cancer (*n*)	Controls (*n*)	Characteristic microbiome	Changes in the cancer group (↑: Enrich; ↓: Reduce)	
Czech Republic	Urinary	63	\	\	34	29	*Veillonella*, *Varibaculum*	↑	134
							*Pasteurellaceae*-related bacterium, *Corynebacterium*	↓	
America	Urinary	53	29	14	43	10	Phylum level: *Actinobacteria*, *Proteobacteria*	↑	124
							Genus level: *Actinomyces*, *Achromobacter*, *Brevibacterium*, *Brucella*		
							Phylum level: *Firmicutes*, *Deinococcus-Thermus*	↓	
							Genus level: *Salinococcus*, *Jeotgalicoccus*, *Escherichia-Shigella*, *Faecalibacterium*, *Thermus*, *Lactobacillus*		
China	Urinary	24	\	\	24	0	Phylum level: *Proteobacteria*, *Firmicutes*, *Actinobacteria*, *Tenericutes*, *Bacteroidetes*	↑	135
							Class level: *Gammaproteobacteria*, *Bacilli*, *Actinobacteria*, *Mollicutes*, *Bacteroidia*, *Betaproteobacteria*, *Clostridia*		
							Order level: *Enterobacteriales*, *Lactobacillales*, *Mycoplasmatales*, *Actinomycetales*, *Xanthomonadales*, *Clostridiales*, *Bacillales*, *Bacteroidales*		
China	Urinary	55	10	19	29	26	Genus level: *Actinomyces*	↑	110
							Genus level: *Streptococcus*, *Bifidobacterium*, *Lactobacillus*, *Veillonella*	↓	
Croatia	Urinary	23	\	\	12	11	*Fusobacterium*, *Actinobaculum*, *Facklamia*, *Campylobacter*, *Subdoligranulum* (94otu11945), *Ruminococcaceae_UCG-002* (94otu9391)	↑	136
							*Veillonella*, *Streptococcus*, *Corynebacterium*	↓	
America	Urinary	37	12	15	27	10	(MIBC) *Bacteroides*, *Faecalbacterium*	↑	119
							*Bacteroides*, *Lachnoclostridium*, *Burkholderiaceae*	↓	
China	Urinary	98	51	0	51	47	(NMIBC) *Eubacterium* sp. *CAG:581*, *Bacteroides* sp. *4_3_*47FAA, *Flavobacteriales*	↑	123
							*Clostridium*, *Clostridiaceae*, *Alistipes*	↓	
							*domain Bacteria*, *Clostridium cluster XI*, *Prevotella*	↓	
China	Fresh fecal samples	47	26	6	32	15	*Streptococcus*, *Escherichia*	↑	137
							*Bifidobacterium*, *Lactobacillus*	↓	
Spain	Tumor and non-tumor samples	58	0	32	32	26	*Barnesiella*, *Parabacteroides*, *Prevotella*, *Alistipes*, *Lachnospiracea_incertae_sedis* (cluster 1), *Staphylococcus* (cluster 2)	↑	116
							*Actinobacteria*	↓	
China	Tumor and non-tumor samples	34	5	17	22	12	Phylum level: *Proteobacteria*	↑	138
							Genus level: *Acinetobacter*, *Escherichia-Shigella*		
							Genus level: *Lactobacillus*, *Prevotella_9*	↓	

### Therapeutic value of microbiota in BLCA

In recent decades, BLCA treatment strategies have evolved significantly. Beyond traditional radical surgery and chemotherapy, immunotherapies like oncolytic HSV, intravesical BCG perfusion, and immune checkpoint inhibitors (ICIs) have been introduced. These treatments are particularly beneficial for patients who do not respond well to standard first-line options. Immunotherapy currently benefits a limited number of patients with BLCA, with many experiencing adverse effects. Future improvements could revolutionize treatment by enhancing response rates and managing side effects.^140^ In recent years, some studies have found that microbiota not only affect the occurrence and development of BLCA but are also related to the efficacy of BLCA immunotherapy.^141^, ^142^, ^143^, ^144^

### Role of oncolytic HSV in BLCA therapy

Oncolytic HSV can trigger various cell death signals and release multiple damage-associated substances, thereby directly and indirectly exerting its oncolytic effects through the release of tumor antigens.^145^^,^^146^ In recent years, several clinical trials have evaluated the safety and efficacy of oncolytic HSV therapy in patients with BLCA, with some showing favorable clinical responses. Researchers have genetically modified HSV to enhance its selective ability to kill cancer cells and to stimulate the patient's immune response, thereby increasing the anti-tumor effect.^147^^,^^148^ Oncolytic HSV treatment is often combined with other therapies, such as immunotherapy or chemotherapy, to improve overall efficacy.^149^ Although the overall safety profile of oncolytic HSV therapy is relatively high, monitoring and management of potential side effects are still necessary to ensure patient safety and the acceptability of the treatment.

### The role of microbiota in intravesical BCG therapy

Hussein et al^124^ found that *Serratia*, *Brochothrix*, *Negativicoccus*, *E. coli-Shigella*, and *Pseudomonas* were significantly more abundant in the urine of patients with NMIBC who responded to BCG perfusion therapy than in non-responders. BCG has been pivotal in treating NMIBC, effectively reducing tumor recurrence and progression.^150^ However, the role of BCG in the development of MIBC or BLCA recurrence is complex. Current evidence suggests that BCG does not directly cause MIBC, as progression often relates more to tumor biology and patient factors than to the therapy itself.^151^ Some patients may already have aggressive disease that advances despite treatment, and high-grade invasive disease can sometimes be masked by inadequate staging. Thus, any potential link between BCG and progression should not be viewed as a direct causative relationship. Additionally, the connection between the enrichment of these bacterial genera and the therapeutic effects of BCG warrants further investigation.

### Role of gut microbiota in ICI therapy

Mager et al^152^ found that gut microbes like *Bifidobacterium*, *Lactobacillus*, and *Olsonella* enhance tumor immunotherapy by producing inosine, significantly improving the efficacy of ICIs. In addition, vitamin D modifies the microbiota to promote *Bacteroides fragilis* growth, enhancing cancer immunity and leading to stronger immune-dependent resistance to transplanted tumors and better responses to ICI therapy in mice.^153^ Most of these studies have been conducted in a pan-cancer setting, and it is not known whether a similar situation exists in BLCA. Pederzoli et al^154^ conducted a study on the association between fecal microbiota profiles and the response to the neoadjuvant pembrolizumab in patients with MIBC and found differences in the enrichment of 16 bacterial taxa between responders and non-responders. *Sutterella* was enriched in responders, whereas *Ruminococcus bromii* was enriched in non-responders, and the negative effect of *R. bromii* on anti-PD-1 antibody activity was also observed in a mouse model. However, further studies are needed to verify the relationships between bacterial taxa and response and to elucidate the specific mechanism of action. Pederzoli et al^155^ found that antibiotic treatment, particularly fluoroquinolones, reduced the efficacy of immunotherapy in patients with MIBC, leading to lower rates of pathological complete response and higher recurrence rates, potentially due to disruptions in intestinal microflora.

### Role of urinary microbiota in ICI therapy

Previous studies^156^ have also shown that BLCA recurrence and progression involve the PD-L1/PD-1 pathway, and evaluation of drug responsiveness is necessary before immunotherapy targeting PD-1/PD-L1 in BLCA. Current methods for predicting treatment response, like assessing PD-L1 expression for pembrolizumab efficacy, are limited, as many PD-L1-positive patients still do not respond well to immunotherapy.^157^ Therefore, identifying additional biomarkers is essential to better assess the suitability of immunotherapy in PD-L1-positive patients, helping to avoid adverse reactions and unnecessary costs. Chen et al^158^ found that urinary microbiota richness correlates positively with PD-L1 expression in male patients with NMIBC, suggesting that urinary flora may influence BLCA recurrence and PD-L1-targeted immunotherapy efficacy, indicating its potential as a predictive biomarker, though further research is needed. Bi et al^110^ examined the difference in microbiota between MIBC and NMIBC. At the phylum level, the composition of the dominant bacteria (*Proteobacteria*, *Firmicutes*, and *Actinobacteria*) in the two groups was similar, and all routinely colonized the urine. In contrast, the abundances of *Bacteroidetes*, *Firmicutes*, and *Firmicutes_A* were significantly lower in MIBC than in NMIBC. Based on the random forest analysis, *Ralstonia* sp*000620465*, *Cutibacterium acnes*, and *Ralstonia pickettii* were the three most important species between the MIBC and NMIBC groups. *Herbaspirillum*, *Porphyrobacter*, and *Bacteroides* were enriched in patients with BLCA with a high risk of recurrence and progression. These findings suggest that targeting the microbiota in different BLCA subtypes is a feasible therapeutic strategy, potentially enhancing immunotherapy responses by altering the tumor immune ecology.

### Role of intratumoral microbiota in immunotherapy

In addition to the gut and urinary microbiota, the intratumoral microbiota is also involved in the regulation of tumor immunotherapy responses. Yao et al^159^ found that the *Mycoplasma* and *Streptomyces* genera were enriched in patients with BLCA with a durable response to BCG therapy, suggesting that the intratumoral microbiota may enhance the immunotherapy response of BLCA. However, one study^160^ found that *Fusobacterium nucleatum* was ubiquitous in breast cancer and created an immunosuppressive TME characterized by high myeloid cell infiltration, which impedes the efficacy of ICI therapy. In addition, a pan-cancer analysis^161^ of the microbiota of metastatic cancer indicated that the presence of *Fusobacterium* was associated with poor response to ICI treatment in patients with non-small cell lung cancer. *Fusobacterium nucleatum* is a Gram-negative, anaerobic oral commensal bacterium commonly found in the human oral cavity and occasionally in the lower digestive tract of healthy individuals. Recent studies have shown that *F. nucleatum* is not only limited to oral problems, but is also related to the occurrence and development of a variety of cancers. Some studies have reported the presence of *F. nucleatum* in BLCA tissue samples; however, its specific role in the development of BLCA and its effect on the TME and response of the host immune system need to be further studied.^131^

## Clinical application of microbiota in BLCA

### Mouse model construction for microbiota research

The preclinical studies of BLCA primarily utilize mouse models through four main approaches (Fig. 4): BLCA cell line transplantation, chemical carcinogen induction, genetic engineering, and patient-derived xenografts. Cell line-based subcutaneous implantation remains the most common method due to its simplicity and cost-effectiveness. To better mimic the bladder microenvironment, tumor cells are pretreated with enzymatic or chemical methods (*e.g.*, trypsin, electrocoagulation) before intravesical delivery via urethral perfusion, abdominal incision, or ultrasound-guided techniques.^162^, ^163^, ^164^, ^165^ Genotoxic chemicals like N-butyl-N-(4-hydroxybutyl) nitrosamine and 20-methylcholanthrene induce BLCA by damaging bladder urothelial DNA.^164^ Genetically engineered mouse models use bladder-specific promoters (*e.g.*, UpkII) to study gene effects. UpkII-driven SV40T causes carcinoma, H-Ras leads to hyperplasia, and combined SV40T/H-Ras induces invasive carcinoma within one month.^166^ Cre-LoxP systems enable controlled gene manipulation via adenoviral delivery.^167^ Patient-derived xenografts involve transplanting BLCA tissues into NSG mice, requiring 2–6 months for establishment. The patient-derived xenograft retains 92%–97% the genetic changes and morphology of the original tumors, supporting drug testing and immunotherapy studies.^168^

**Figure 4:**
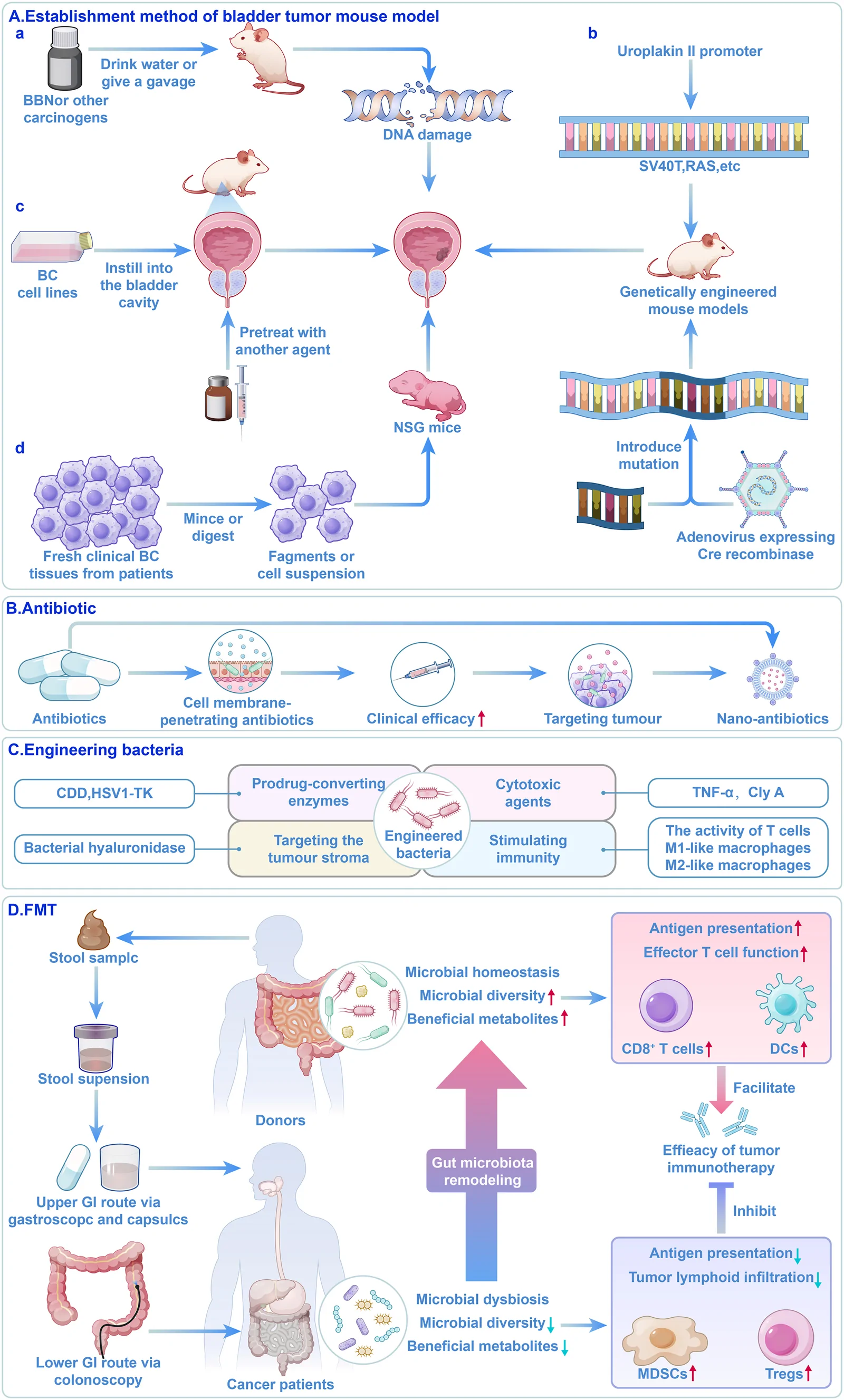
Strategy for establishing a mouse model for bladder cancer treatment. **(A)** Mouse model construction for microbiota research. (a) Mouse bladder model pretreated with physical or chemical methods. Tumor cells are inoculated into the bladder through a transurethral catheter or surgery. (b) The genotoxic chemical is administered to mice through drinking water or gavage, causing DNA damage to the bladder and then inducing bladder cancer. (c) By knocking in or out specific genes, gene recombination can be selectively achieved in the bladder epithelium to construct a genetically engineered mouse model. (d) Tumor tissues from patients with bladder cancer were chopped to an appropriate size and transplanted into immunodeficient mice to construct patient-derived xenotransplantation mouse models. **(B)** Antibiotics enhance the efficacy of tumor treatment and lead to the development of more targeted nano-antibiotics for tumors. **(C)** Engineering bacteria serve four key functions in cancer therapy: they act as prodrug-converting enzymes to enhance drug activation, target the tumor stroma to improve drug delivery, produce cytotoxic agents to directly destroy tumor cells, and stimulate immune responses to boost the body's defense against cancer. **(D**) Fecal microbiota transplantation is closely associated with the modulation of immune responses, enhancing the effectiveness of immunotherapy in cancer treatment.

Each of these methods has advantages and disadvantages. For example, it is easier to access the bladder via the urethra in female animals, but this introduces a sex bias to the model. Perfusion through an abdominal incision carries the risk of infection. Exposure to chemicals that are directly or indirectly carcinogenic via *in vivo* metabolism is widely used to induce BLCA. These chemicals are often genotoxic and can cause DNA damage in bladder urothelial cells in mice when ingested via drinking water or gavage, thereby inducing BLCA. N-butyl-N-(4-hydroxybutyl) nitrosamine is one of the most common carcinogens. Other commonly used chemicals include amine compounds such as N-nitrosamine, anthracene compounds such as 20-methylcholanthrene, and N-[4-(5-nitro-2-furyl)-2-thioacyl-]formamide.^167^ In genetically engineered mouse models,^166^ knock-in or knockout mice can be used to study the effects of alterations in specific genes on cancer. The most commonly used promoter driving bladder-specific expression of target genes is the mouse UpkII promoter. Under the regulation of the UpkII promoter, SV40T antigen expression leads to the development of low-copy number carcinoma *in situ* and high-copy number invasive metastatic transitional carcinoma, and H-Ras expression leads to urothelial hyperplasia in low-grade papillary BLCA. Double-transgenic mice carrying SV40T and H-Ras can develop high-grade invasive urothelial carcinoma within one month, although such mice die at approximately seven weeks of age.^168^ Patient-derived xenografts involve collecting fresh clinical BLCA tissue from a patient and mincing it into 3–5 mm^3^ pieces, and tumor tissues can also be digested to a cell suspension and implanted into the bladder wall to establish an *in situ* bladder carcinoma model.^169^

### Antibiotic and engineered bacteria

Recently, antibiotic applications and engineered bacteria have garnered attention as potential avenues for enhanced treatment modalities. Antibiotics, traditionally employed to combat bacterial infections, have shown promise in modulating the TME and enhancing the efficacy of immunotherapies. Certain antibiotics can alter the microbiome, impacting immune responses and patient outcomes.^170^ Additionally, targeting tumor bacteria to expose microbial epitopes may improve cancer treatment.^171^ Engineered bacteria, particularly those designed for targeted drug delivery or immune modulation, have emerged as a groundbreaking approach in cancer therapy.^172^^,^^173^ These bacteria can be genetically modified to express specific therapeutic agents or to selectively target tumor cells while sparing healthy tissues, minimizing side effects.^174^^,^^175^ Moreover, they can be utilized to transform immunologically “cold” tumors into “hot” ones, thereby enhancing treatment efficacy.^176^ Overall, these innovative therapeutic modalities offer a promising frontier in the battle against BLCA.

### Fecal microbiota transplant

Roje et al^177^ have proposed that microbial biotransformation of environmental carcinogens is linked to bladder cancer development, providing new mechanistic insights that could enhance microbiome-targeted strategies for cancer risk assessment and prevention. Multiple studies have used fecal microbiota transplant or bacterial isolates to improve the efficacy of immune checkpoint-blocking therapies.^178^, ^179^, ^180^, ^181^ Fecal microbiota transplant has shown promise alongside checkpoint-blocking therapy, as germ-free mice reconstituted with fecal material from patients with responsive cancer exhibited a similar response to PD-1 blocking, while those reconstituted with material from unresponsive patients did not respond, a phenotype that can be reversed by fecal microbiota transplant from immunoresponsive patients.^182^ In addition, anti-PD-1 efficacy appears to be reduced in patients receiving broad-spectrum antibiotics.^181^ Bacteria-based tumor-targeted therapy remains a promising application, providing many possibilities for tumor treatment. Canale et al^183^ developed a strain of *E. coli* (Nissle 1917) using synthetic biology methods, which colonized tumors and increased the number of tumor-infiltrating T cells by continuously converting ammonia to l-arginine. It also has a significant synergistic effect with PD-L1 blocking antibodies on tumor clearance.

### Challenges and limitations of BLCA microbiota

There remain many challenges in studying the interactions between microbiota and BLCA, including the acquisition of specimens, limitations in detection techniques, identification of the causal relationship between microbiota and tumors, and heterogeneity among individuals.

### Sampling is prone to contamination

The amount of urine and tumor microbes is small compared with gut microbes, necessitating microbial enrichment while preventing contamination. Contamination usually arises from sample collection, treatment, and analysis, with the collection process being the most vulnerable due to the varying methods and lack of standardization. Various urine collection methods introduce different microbial confounding factors, including midstream collection, catheter collection, suprapubic bladder puncture, and cystoscopic drainage. Midstream urine is easily contaminated by urethral, genital, and skin microbiota.^184^ Cystoscopy and catheter drainage of urine are also easily contaminated by microbiota in the urethra and urethral orifice. Suprapubic bladder puncture drainage can reflect the actual microbial situation when there is a sterile puncture.^185^ Current sampling methods for intestinal microbiota need improvement. The fecal swab method is popular due to its non-invasive, convenient, and cost-effective nature, but bacterial distribution varies in the digestive tract, making fecal content differ significantly from that in the lower digestive tract. Other methods, like colonoscopy, increase costs and may be unacceptable to healthy subjects. Therefore, developing a more convenient, economical, and less invasive approach for lower digestive tract sampling is essential for standardization. For tumor microbiota, despite high surgical sterility, contamination risks remain during specimen acquisition, storage, redistribution, and inspection.^186^ It is crucial to control whether tumor specimens contain normal adjacent tissue, to assess differences between surgical approaches, and to regulate preservation conditions for aerobic/anaerobic bacteria.

Microbiota extracted from urine, feces, or surgical specimens reflect only a specific individual's microbial composition at one point in time. The complex and dynamic interactions between humans and microbes mean that laboratory animal models cannot fully replicate human microbiota. Furthermore, we still lack an understanding of how various microbiota influence BLCA and how factors like abundance, enrichment duration, and survival affect it.

### Limitations of detection technology

The current mainstream method for microbiota sequencing is 16S rRNA gene sequencing. The advantages of this detection technology have been discussed above; however, this method faces several challenges.

The 16S rRNA detection technique cannot differentiate between live and dead bacteria or identify microbiota with thick cell walls. Therefore, expanded quantitative urine culture is often used as a complementary method. It can not only detect common urinary pathogens but also emerging pathogens and symbiotic flora.^187^ In addition, 16S rRNA copy numbers make it difficult to accurately assess the relative abundance of species. Intragenomic heterogeneity of 16S rRNA genes has been reported in many studies and biases estimates of prokaryotic biodiversity.^188^ Variations in the 16S rRNA gene sequence within the same strain can occur, complicating absolute quantitative analysis and leading to biased diversity assessments.^189^ The inability of the 16S rRNA method to distinguish closely related bacterial taxa poses challenges. Changes in the relative abundance of a specific microbiota group may not solely result from its own abundance variation, as changes in other groups can also influence these ratios.^190^ Accurately and quantitatively analyzing microbial community diversity remains a challenge.

### Temporal and spatial heterogeneity of microbiota

A major challenge in clinical research is microbiota heterogeneity, as each individual's unique composition complicates generalization from limited samples. Variations in microbiota across populations and detection methods hinder reproducibility, making it difficult to draw broad conclusions, given that composition can vary between and within individuals over time.^191^ Whether microbial plasticity can explain how individuals with the same susceptibility traits develop unique disease characteristics should be considered. While microbiota variability exists over time within individuals, there is still stability in community structure, reflecting “elastic” dynamic changes in the microbiota. For example, in the gut microbiota, this dynamic change is manifested in the ability to return to baseline levels after a brief disruption of homeostasis (*e.g.*, antibiotic exposure)^192^ and diurnal and highly repetitive microbiota changes in healthy gut microbiota over a 24-h period.^193^

More research is needed to further validate the unique microbiota profiles present in populations of diverse geographic, ethnic, and cultural backgrounds to provide holistic treatment guidelines for the individualized treatment of BLCA.

### Causality between microbiota and BLCA

Most studies on microbes and BLCA are observational, showing only associations. Causality is complex and uncertain, particularly regarding interactions between microbiota and tumors.

Several experimental approaches have attempted to infer a causal relationship between microbiota and bladder tumors; these include fecal microbiota transplant, human microbiota-associated mouse models, and microbial phenotyping triangulation. In short, fecal microbiota transplant is a treatment in which feces are collected from a tested healthy donor and microbes are transplanted into the patient via colonoscopy enema or capsule form;^182^ human microbiota-associated mouse models are the ones that similarly transfer human gut microbiota into mice.^194^ The former observes disease outcomes after microbiota interventions, while the latter studies human gut microbiota interactions in mice post-transplantation. The microbial phenotyping triangulation process involves analyzing diverse data to identify recurring associations between microbiota and health conditions, allowing for more confident causal inferences through “triangulation” of multiple perspectives. Cross-disciplinary research in biomedical and social sciences can bridge laboratory and patient-level applications, address gaps in BLCA-related microbiota research, and encourage re-evaluation of foundational hypotheses for more flexible causal approaches. Standardized animal models and advanced microbial research tools can enhance the accuracy of findings.

## Conclusions and perspectives

The exploration of BLCA-related microbiota is still in its infancy due to detection technology limitations and other factors. While these challenges hinder understanding of the relationship between microbes and BLCA, they do not diminish the optimistic outlook for future research in this area.

Further exploration of the BLCA microenvironment using microscopy and individualized *in vitro* models is essential. Artificial intelligence (AI) methods can facilitate large cohort data collection and deep multivariate analyses of microbiota and personal characteristics, leading to reliable AI models. This integration will enhance precise, individualized, and data-driven diagnosis and treatment of BLCA. AI models can also aid in inferring immunotherapy effectiveness and predicting survival, while considering the spatial relationship between tumors and microbiota. Imaging-based spatial transcriptomics allows measurement of the RNA distribution in single cells, facilitating gene expression profiling.^195^ This technique can reveal how microbiota affect the TME by linking host cell pro-tumor expression to microbiota structures. Niño et al^196^ identified microbial species in patient tissues and their corresponding localization in host cells and induced related molecular changes, which provides a foundation for spatial transcriptomics to study the role of the microbiota in the TME.

Mouse models are essential for studying host–microorganism interactions in BLCA microbiota, but creating models that accurately replicate human microbiota in a controllable and scalable manner remains challenging. Simplified *in vitro* research methods, such as human organoids^197^ and microfluidic-based organ-on-a-chip models,^198^ have been developed, which provide new possibilities for studying the interactions of microbiota and BLCA *in vitro*. Moreover, organoid and chip-organ models utilize patient tissue, and with reduced costs, individualized patient models may soon be developed to evaluate drug therapy efficacy.

In conclusion, despite ongoing challenges in studying BLCA-related microbiota, advancements in detection methods and interdisciplinary research hold promise for improving the prevention, detection, diagnosis, and treatment of BLCA by incorporating microbiota.

## CRediT authorship contribution statement

**Zhihang Luo:** Writing – original draft, Visualization, Investigation, Formal analysis. **Daming Xu:** Investigation, Writing – review & editing, Visualization, Formal analysis, Validation. **Zizhuo Wang:** Writing – original draft, Visualization, Investigation. **Longxiang Wu:** Writing – original draft, Investigation. **Chunlin Ou:** Writing – review & editing, Validation, Supervision, Project administration, Funding acquisition, Conceptualization. **Zhenyu Ou:** Writing – review & editing, Validation, Supervision, Project administration, Funding acquisition, Conceptualization.

## Funding

This study was supported by the 10.13039/501100001809National Natural Science Foundation of China (No. 82373062), the 10.13039/501100004735Hunan Provincial Natural Science Foundation of China (No. 2023JJ30929), and the 10.13039/501100002822Central South University Innovation-Driven Research Programme (China) (No. 2023CXQD075, 2023CXQD077).

## Conflict of interests

The authors declared no competing interests.
